# Qualification and Implementation of a Robust Multi-Attribute Method Platform Across Multiple Laboratories for High-Resolution Process Characterization of Biopharmaceutical Products

**DOI:** 10.3390/ph19081147

**Published:** 2026-07-24

**Authors:** François Griaud, Patrick Sascha Merkle, Joachim Ritter, Victor Le-Minh, Haiyan Zhang, Maja Semanjski Curkovic, Stefan Mittermayr, Eva Kranjc, Nina Pirher, Jens Pettelkau, Markus Nienhaus-Stepath, Dominik Mertens, Guillaume Rey, Jérôme Dayer, Manuel Lang, Joanna Hajduk, Camille Jenny, Michel Starck, Thomas Jamnik, Lukas Dotzauer, Dariusz Janecki, Thomas Pohl

**Affiliations:** 1 Technical Research & Development (TRD) Biologics Analytical Characterization, Novartis Pharma AG, 4002 Basel, Switzerland; 2 TRD Biologics Drug Substance Development, Novartis Pharma AG, 4002 Basel, Switzerland; 3 TRD Biologics Process Analytical Sciences, Novartis Pharmaceutical Manufacturing LLC, 1234 Mengeš, Slovenia; 4 TRD Biologics Analytical Characterization, Novartis Pharma, 6250 Kundl, Austria; 5 Genedata GmbH, 80339 Munich, Germany; 6 Genedata AG, 4051 Basel, Switzerland; 7 TRD Biologics Process Analytical Sciences, Novartis Pharma AG, 4002 Basel, Switzerland; 8 TRD Biologics Process Analytical Sciences, Novartis Pharma AG, 106336 Schaftenau, Austria; 9 TRD Biologics Analytical Characterization, Novartis Pharma AG, 82041 Oberhaching, Germany

**Keywords:** multi-attribute method, MAM, mass spectrometry, process characterization, design of experiments, critical quality attribute, critical process parameter, quality target product profile

## Abstract

**Background/Objectives:** Process characterization (PC) of biopharmaceutical products is intended to identify critical process parameters (CPPs) based on their impact on critical quality attributes (CQAs), as well as to define acceptable process parameter ranges to ensure consistent product quality and process performance. However, conventional analytical methods with indirect readouts, e.g., the sum of size/charge variants, often fall short in differentiating CQAs that have safety and efficacy relevance from product quality attributes (PQAs) that do not, thus limiting their utility in establishing a thorough process understanding. The Multi-Attribute Method (MAM) addresses these limitations by monitoring CQAs using the required resolution and specificity. **Methods:** A MAM platform was developed, and its robust performance and reproducibility were demonstrated across six laboratories. The MAM platform was applied for the drug substance (DS) PC of two monoclonal antibodies and the analysis of up to a hundred PQAs across 472 and 644 samples, respectively. The MAM results were compared to data obtained with conventional methods like hydrophilic interaction chromatography–fluorescence detection (HILIC-FLD) and cation-exchange chromatography with UV detection (CEX-UV). **Results:** The levels of PQAs, including succinimide, deamidation, glycosylation, and oxidation, were reproducible between six laboratories. Artefactual oxidation was limited by controlling the quality of TFA reagent. CPPs impacting the level of, e.g., oxidation, glycation, O-glycosylation, and N-glycan sialylation, were identified, leveraging a simultaneous, consistent, and fast MAM data analysis across all samples. **Conclusions:** MAM enables high-resolution PC to identify CPPs and inform control strategies for CQAs that are not resolved by conventional methods.

## 1. Introduction

Quality by design (QbD) represents a systematic approach to development that begins with predefined objectives and emphasizes product and process understanding and process control, which is based on sound science and quality risk management [1]. The main objective of QbD is to comprehensively identify critical quality attributes (CQAs) and control their variability throughout the entire supply chain to ensure a safe and efficacious product at the time of administration. This requires linking critical material attributes (CMAs) and critical process parameters (CPPs) to CQAs to establish a robust control strategy, ensuring that the final product consistently meets the predefined quality target product profile (QTPP) [2].

Chemical or post-translational modifications (PTMs) of a protein biopharmaceutical, such as deamidation, isomerization, glycation, clipping, or N-/O-glycosylation, may be classified as CQAs based on their impact on biological activity, pharmacokinetics/pharmacodynamics, immunogenicity, or safety [3]. Once CQAs are identified, the process characterization (PC) exercise aims to identify CPPs and define their acceptable ranges based on their impact on CQAs [2]. However, inaccurate CQA monitoring and an inadequate control strategy may result from applying analytical methods lacking specificity and resolution to determine the impact of the process on a CQA. For example, several product-related variants may co-elute in, e.g., size/charge variant analyses, e.g., by capillary electrophoresis sodium dodecyl sulfate (CE-SDS), cation-exchange chromatography (CEX), capillary zone electrophoresis (CZE), or N-glycan profiling by hydrophilic interaction chromatography (HILIC), thus limiting process understanding of the influence of process parameters on, e.g., the “sum of acidic/basic variants”, “sum of fragments”, or “main variant”. In conclusion, a control strategy relying on indirect measurements such as the sums of variants may not be sensitive or specific enough to the CQA, thus limiting flexibility in the commercial lifecycle due to a high number of CPPs, narrowing the process parameter ranges, and, at worst, compromising product safety and efficacy.

The Multi-Attribute Method (MAM) is being employed across the biopharmaceutical industry to monitor products’ CQAs in pharmaceutical development and manufacturing [4,5,6,7]. MAM is based on a reduced peptide-mapping workflow that allows monitoring of individual chemical modifications on the polypeptide chain of the protein biopharmaceutical. Sample preparation parameters have been comprehensively reviewed to limit method artefacts, such as deamidation and oxidation, or enzymatic missed cleavage [4,8,9,10,11,12,13,14]. Following an in-depth characterization of product-related variants by liquid chromatography coupled to tandem mass spectrometry (LC-MS/MS), a project-specific MAM peptide library can be built and applied for routine testing by LC-MS [5,6,7,8]. MAM enables consistent reporting of low-abundance attributes that might not be selected for MS/MS or PTMs that are unmatched with a search engine in classical peptide-mapping workflows [15]. LC-MS instrumentation has been shown to successfully detect low-level sequence variants in the range of 0.01–0.5% in biopharmaceutical products where conventional analytics remain limited in resolution and sensitivity [16,17]. Leveraging its sensitivity, specificity, and attribute multiplexing capabilities, MAM may replace conventional analytical techniques during stability testing, batch release, and process analytics [5].

However, repetitive MS data processing and data analysis/review steps appear to be limiting performance factors when scaling up MAM to enable parallel monitoring of multiple attributes in large sample cohorts generated during a PC exercise. While the majority of previously reported studies were rather restricted to smaller sets of quality attributes and a relatively low number of samples [10,11], only a recent report showcases the application of a fast sample preparation step and highly automated MAM data acquisition and analysis workflow to study large sample sets [18]. Therefore, aside from a fast sample preparation step, a streamlined MS data analysis and review workflow that enables fast detection and the quantification of multiple PQAs across several hundred samples is required to extend the use of MAM to PC in a time-efficient manner.

In this work, we showcase the robust performance of a generic MAM platform, implemented across six development laboratories, notably by limiting the introduction of sample preparation-related artefacts for the accurate monitoring of labile and sensitive PQAs such as succinimide formation, isomerization, deamidation, and oxidation. We discuss the challenges and solutions related to the deployment of MAM at multiple laboratories, including the implementation of a retention time recalibration tool and the control of critical reagents to limit oxidation artefacts. The platform was used to enable simultaneous data processing of up to a hundred PQAs in large sample cohorts of up to 600 samples in a week, thus offering substantial time savings to support upstream (USP) and downstream (DSP) process characterization Design-of-Experiment (DoE) studies of two monoclonal antibodies (mAbs). These PC studies provide a comprehensive understanding about the influence of process parameters on CQAs, such as N-glycosylation (including high-mannose, galactosylation, and sialylation species), glycation, oxidation, deamidation, and O-glycosylation in complementarity-determining regions (CDRs). Accordingly, this study highlights the capabilities of MAM to identify CPPs and delineate acceptable ranges to control site-specific CQAs during the PC exercise of biopharmaceutical products, where conventional CEX-UV and HILIC-FLD lacked the required specificity and resolution due to the co-elution of multiple variants under a single chromatographic peak.

## 2. Results

### 2.1. MAM Platform Robustness and Inter-Laboratory Performance

**Figure 1:**
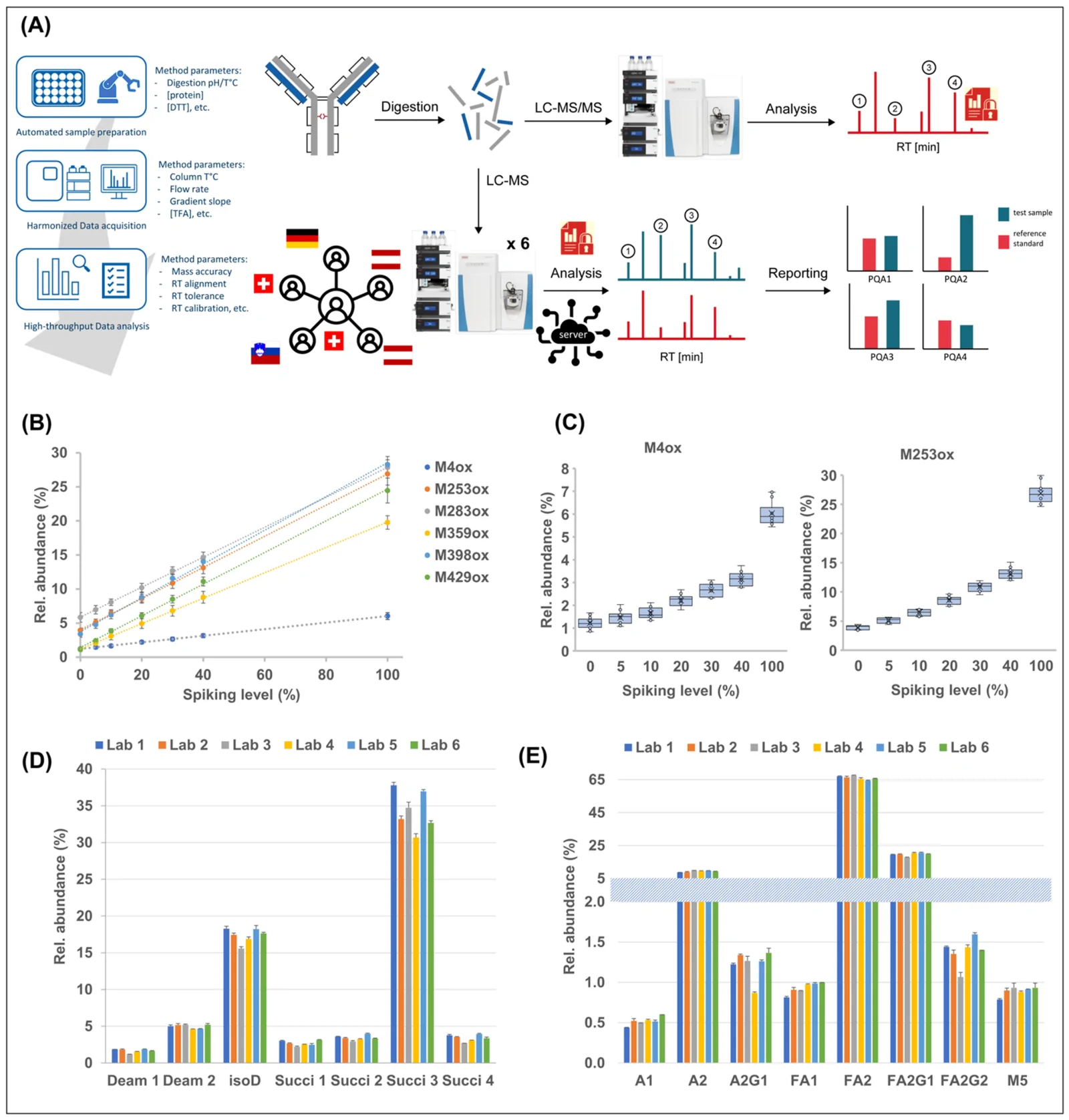
MAM platform and inter-laboratory performance across six laboratories. (**A**) Presentation of the MAM platform including automated sample preparation, harmonized data acquisition, and high-throughput data analysis. Data were processed at each site using the same data analysis workflow available via a server application. Circled numbers indicate different PQAs. (**B**) Linearity of all methionine (M) oxidation sites across all six laboratories (*n* = 18). Error bars display the standard deviation. (**C**) Inter-laboratory reproducibility for the low abundant oxidation at methionine 4 (M4ox) and a higher abundant oxidation at methionine 253 (M253ox) for each stress level (*n* = 18). Plots display the median and the first and third quartile, while error bars display the minimum and maximum, respectively. (**D**) Inter-laboratory reproducibility for labile chemical modifications at several sites (*n* = 3). Error bars display the standard deviation. Succ: succinimide; isoD: iso-aspartate; Deam: deamidation. (**E**) Inter-laboratory reproducibility for N-glycopeptides with monoantennary (A1), biantennary (A2) and high-mannose (M5) N-glycans (*n* = 3). Error bars display the standard deviation. F: fucosylation; G1: 1 galactose moiety; G2: 2 galactose moieties. The dashed area separates different scales of the y-axis.

The MAM platform used in this study is composed of the following three components: fully automated sample preparation on a liquid handling system, harmonized LC-MS instrumentation for data acquisition and high-throughput data analysis on a server application (Figure 1A). In-depth characterization is performed using an LC-MS/MS workflow to build project-specific PQA libraries, as described previously [6,8]. The minimum information contained in these libraries is the PQA name, associated with their specific mass-to-charge (*m*/*z*) ratios and retention times (RTs). Once established, such project-specific PQA libraries can be applied for routine LC-MS monitoring of up to a hundred site-specific PQAs (Figure 1B). Peptides are separated by reversed-phase UPLC coupled to a high-resolution mass spectrometer. Finally, a semi-automated data evaluation workflow enables comprehensive data processing, review and reporting.

The robustness of the MAM platform was evaluated (Figure 1A). In this study, the impact of minor, deliberate variations in sample preparation and LC-MS parameters on the reportable results, i.e., relative PQA abundance, and method performance indicators such as digestion efficiency and cysteine alkylation were investigated. Given the large number of method parameters and method outputs included in the robustness assessment, a DoE approach was adopted to identify the main method parameters that impact method performance and to confirm robust operating conditions while reducing the number of experiments [19,20,21].

MAb1 PQAs including succinimide and isomer formation at aspartic acid residues, deamidation at asparagine residues, N-glycosylation and methionine oxidation were monitored (Table A1). Protein concentration, denaturation pH, DTT concentration, and particularly digestion pH showed significant impacts on the variability of PQAs, such as oxidation, deamidation and succinimide formation (Table A2). Overall, increasing the method parameters led to higher levels of most PQAs, while DTT concentration had an inverse effect. Additionally, a higher degree of variability was observed with low-abundance PQAs. The pH of denaturation and digestion buffers appeared to be the most impactful factors, showing an inverse effect on the sample preparation performance indicators such as cysteine alkylation and missed cleavage, while DTT concentration showed a positive effect on cysteine alkylation (Table A2). Incubation temperature was studied separately as it required modifications to the script of the automated sample preparation. The impact of a minor change in incubation temperature had only a neglectable impact on succinimide levels with relative standard deviation (RSD) < 15%, indicating robust performance under routine operating conditions [22]. Statistical simulations derived from the DoE model demonstrated that the predicted RSD was below 15% for highly abundant PQAs (e.g., ≥4.0%), suggesting robust method performance across the allowable range of sample preparation parameters (Table A3). On the contrary, the variability exceeded an RSD of 20% for PQAs present at levels below 4.0% (e.g., deamidation at NG motif, succinimide intermediate at DG motif); however, this is considered acceptable based on the predicted absolute difference of 0.2% to 0.8% (Table A4). The final TFA concentration used during the quenching did not show any significant impact on the robustness assessment. Acid-catalyzed deamidation in an autosampler at 5 °C has already been reported to be dependent on the concentration of the acidic modifier, e.g., the level of TFA, and thus the pH of the sample solution [23]. We previously observed that reducing the concentration of the TFA stock solution, i.e., from 50% to 10%, to reach the same final concentration of 0.05% was sufficient to avoid artefactual deamidation for long sequences.

The RSD of most PQAs remained below 15% within allowable ranges of LC-MS parameters regardless of relative PQA abundance (Table A4), except for the oxidation of one methionine residue (M283ox) exhibiting an RSD of 34.8%. The column temperature and especially the concentration of trifluoroacetic acid (TFA) in the mobile phase appeared to significantly increase oxidation levels at M283. Of note, TFA quality was not tested during the robustness study but revealed to be a critical factor impacting oxidation as discussed in the context of the mAb2 PC exercise below. Neither the sample preparation parameters nor the LC-MS parameters had an impact on the levels of N-glycosylation variants.

In addition to relative PQA abundance, the retention time of peptides is another important method output, as it directly affects the performance of PQA monitoring. PQAs in the MAM peptide library are matched by their *m*/*z* values and retention times (RTs) within predefined tolerance windows, e.g., 10 ppm and 0.2 min, respectively, to ensure accurate peak detection and avoid false annotations. RT shifts are generally a concern, as they could result in failed PQA detection when significant RT deviations (e.g., >0.2 min) occur compared to expected library values. In general, the assessment of LC-MS parameters revealed excellent reproducibility of the MAM setup regarding peptide retention times. However, minor changes in some LC conditions such as column temperature, flow rate, or gradient slope exhibited a significant impact on peptide elution, as expected. Even within the expected systematic errors for these method parameters, large RT shifts (e.g., >0.2 min) can occur, leading to unsuccessful peak detection by the MAM workflow. Such RT shifts may be corrected by chromatogram alignments or RT recalibration as discussed below.

This MAM platform has been implemented across six laboratories, including non-MS expert laboratories to support the formulation and process development of multiple biopharmaceutical products. To assess the performance of the MAM platform in terms of its linearity, accuracy, precision, limit of quantification (LOQ) and detection (LOD), a ring study across the six laboratories was conducted and data was evaluated based on the ICH Q2(R2) guidance (Figure 1) [22]. Samples with seven different levels of degradation were generated by spiking oxidized and thermally stressed material into an unstressed material. Three independent preparations of each spiking level were prepared centrally, shipped and analyzed at each site in an independent manner using a liquid handling system. Method accuracy is inferred from the linearity data by calculating the recovery of the individual measured values compared to the theoretical value as defined by the linear regression. LOD/LOQ has been evaluated based on the standard error of the y-intercept and the slope of the regression line. Inter-laboratory regression analysis reveals excellent linearity (R^2^ > 0.998), repeatability and reproducibility across a wide range of oxidation levels (Figure 1B,C).

Additionally, excellent reproducibility has been observed for N-terminal pyroglutamate, succinimide, iso-aspartate, deamidation and multiple N-glycans, with RSDs of up to 3.7% for species of relative abundance ≥ 20%, up to 6.9% for species between 3 and 20% relative abundance and up to 7.7% for species < 3% relative abundance (Figure 1D,E). MAM enabled monitoring of high-mannose, monoantennary and biantennary N-glycans attached to a consensus Fc N-glycosylation site. These results show that the MAM platform established at the six independent laboratories is able to provide consistent results across a wide range of abundances for multiple relevant PQAs, including labile chemical modifications. Therefore, the MAM platform is considered fit for purpose for routine drug substance and drug product development support.

### 2.2. Addressing Technical Challenges When Performing MAM at Multiple Laboratories

**Figure 2:**
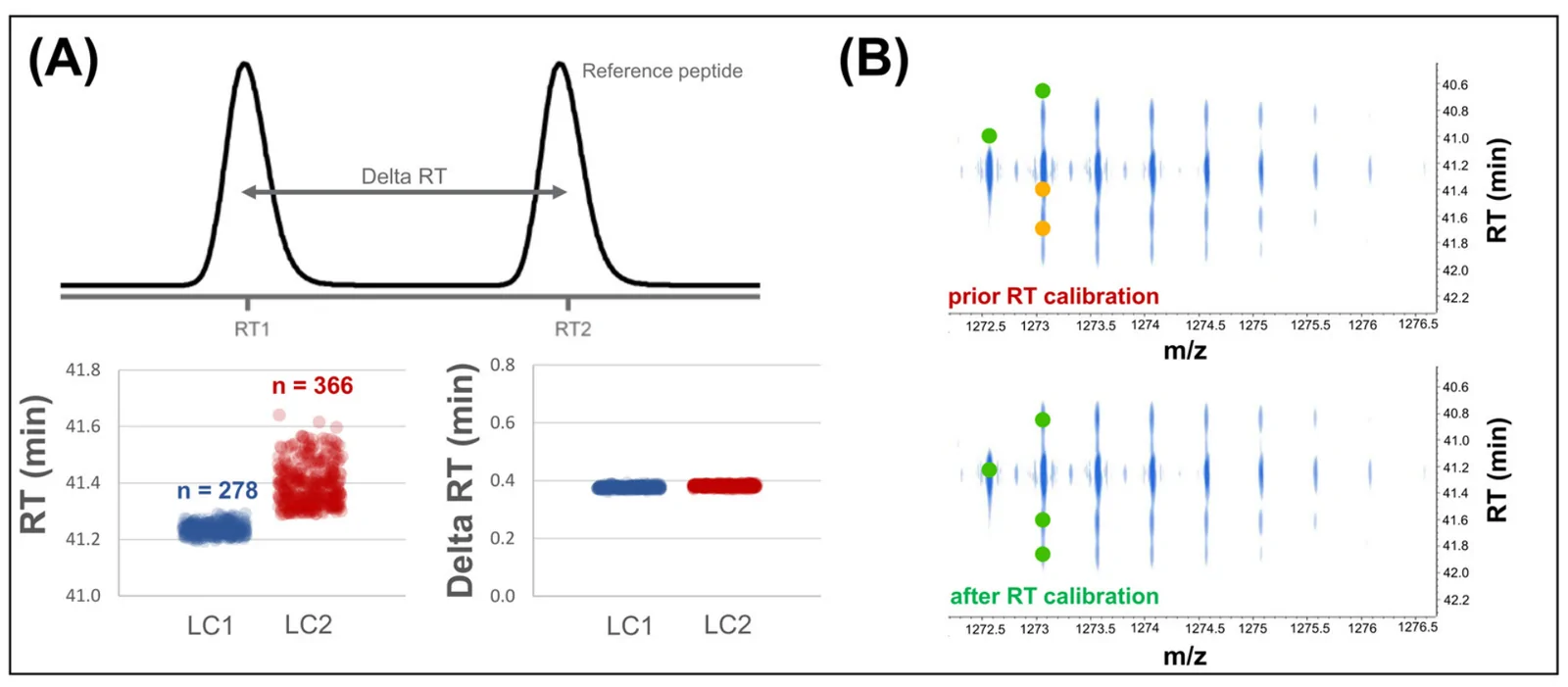
Addressing the impact of chromatographic shifts for MAM analysis at multiple laboratories. (**A**) The RTs of the peptide GFYPSDIAVEWESNGQPENNYK are monitored on the two liquid chromatography (LC) systems and analytical columns before (left) and after automated recalibration (right). For the latter automated recalibration, predefined reference (unmodified) peptides were first annotated with a relaxed RT tolerance window of ≤1.0 min. Differences between the experimental RT values and MAM peptide library RT of these reference peptides were automatically applied to enable the annotation of reference and dependent peptides with stringent RT tolerance window ≤ 0.2 min (see Materials and Methods). (**B**) The validation markers confirm the detection (green), absence (red) or ambiguous assignment (yellow) which is a prerequisite before further data processing and relative quantification. Examples of ambiguous assignments are shown.

Following the reproducibility assessment of the MAM platform at multiple sites, we leveraged two analytical laboratories with identical LC-MS MAM setups, including two robotic systems and two LC-MS instruments, for the analysis of 644 DS process characterization samples of mAb2. The same project-specific MAM peptide library was applied to ensure a systematic and harmonized data analysis process, which was performed independently by each laboratory. As mentioned above, the correction of minor RT shifts is a prerequisite to apply a unique peptide library across multiple laboratories. As shown in Figure 2, the observed RT of the peptide GFYPSDIAVEWESNGQPENNYK (PENNYK peptide) was slightly more variable on the LC system of the second laboratory compared to the first one, with some data points being outside of the RT tolerance window (>0.2 min). Such an RT shift may lead to the ambiguous assignment of deamidated variants on this peptide. After automated recalibration of the RT values of the PENNYK and other predefined reference peptides, minor RT shifts were corrected allowing the unambiguous detection of all product-relevant peptides, including the closely eluting deamidated variants. Validation markers were used to confirm the detection, absence or ambiguous assignments before and after recalibration (Figure 2A), thus facilitating the data analysis. Overall, MAM data processing was streamlined to allow the review and reporting of 172 MAM library features, including unmodified/modified peptides for a total of 77 unique PQAs across 644 samples within one week by one analyst and one reviewer.

**Figure 3:**
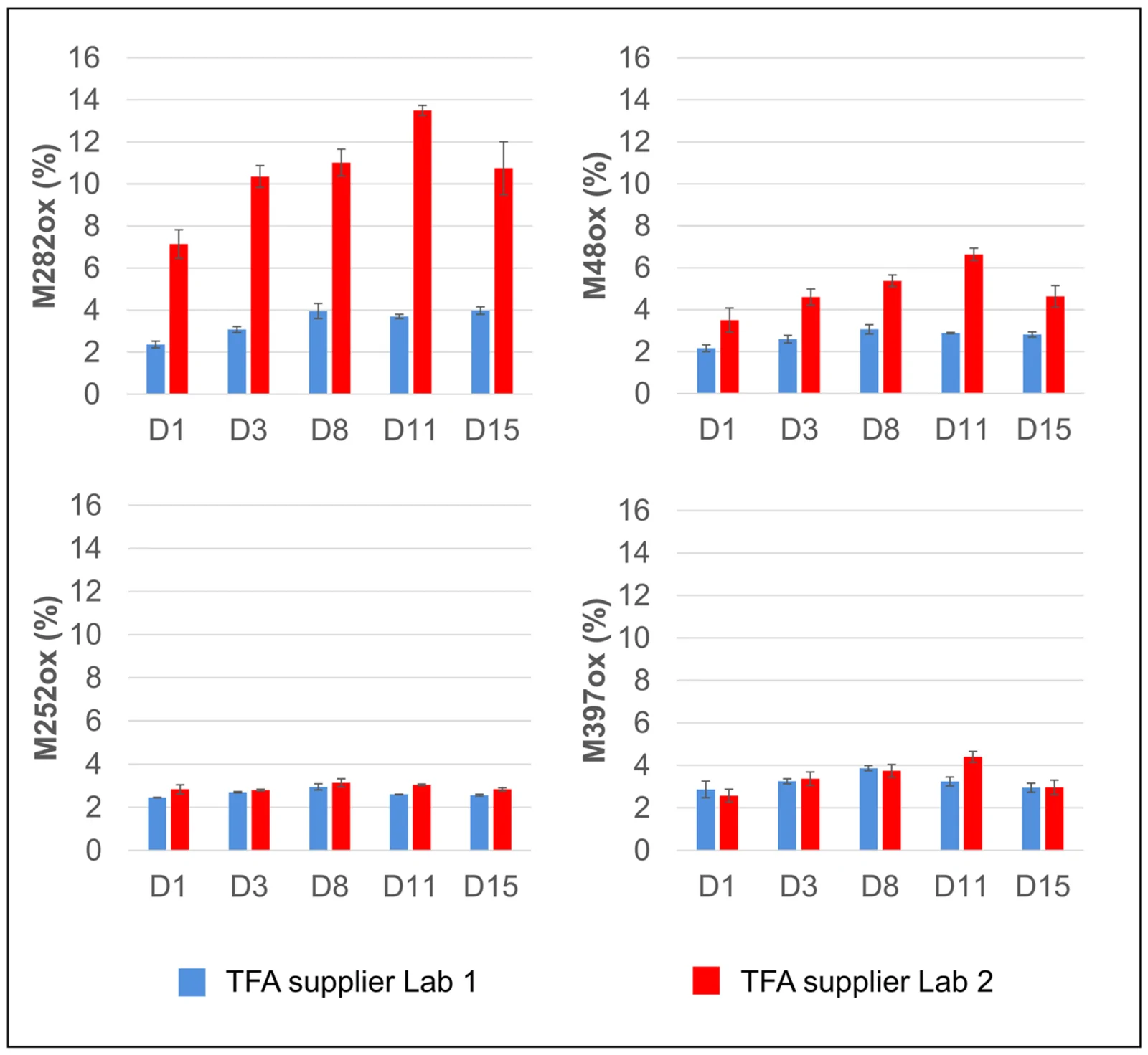
Impact of TFA supplier in controlling artefactual oxidation at multiple laboratories. The oxidation levels at mAb2 heavy chain methionine residues at positions 48, 252, 282 and 397 (M48ox, M252ox, M282ox, and M397ox) are monitored in reference material analyzed with fresh and aged mobile phases from day 1 to day 15 (D1 to D15) in laboratory 1 prepared with TFA from the supplier of laboratory 1 (blue) and TFA from the supplier of laboratory 2 (red). Errors bars correspond to standard deviations of triplicate measurements.

Another challenge encountered during the mAb2 process characterization exercise was the artificial oxidation of methionine residues. Increasing oxidation levels were observed during analytical sequences at one of the two laboratories, especially for mAb2 M282 and M48 in the CDR. A root cause investigation pointed toward different TFA reagent manufacturers used in the two laboratories. Therefore, a confirmatory experiment was performed with reference material in the first laboratory using mobiles phases prepared with the two TFA reagent suppliers. As evident from the reference trending data in Figure 3, oxidation at mAb2 M282 and M48 is dependent on the TFA reagent supplier used and the age of the mobile phase, as higher oxidation was observed with the increasing age of the mobile phase. Interestingly, this effect was not observed at M252 and M397, for which oxidation levels remained constant for 15 days, thus suggesting an influence of the three-dimensional structure or sequence motif prone to potential site-specific interactions with impurities leading to oxidation. As no relevant differences in TFA quality were observed by gas chromatography, we hypothesized that higher levels of elemental impurities may lead to such observations [24,25,26]. Therefore, adequate system-suitability test (SST) criteria should be put in place to control artificial methionine oxidation induced by potential metal contamination originating from TFA reagent quality, aged mobile phases, UPLC capillaries and flow path or glassware washing procedures. These results show the importance of tracking and testing reagents before implementation to ensure consistent MAM workflow performance during long analytical sequences.

### 2.3. MAM to Support DS Process Characterization

Leveraging the MAM platform performance and workflow defined in Figure 1, mAb3 DS PC samples were processed using the automated sample preparation in a 96-well plate format. These samples were obtained from USP and DSP DoEs to identify the CPPs impacting mAb3 CQAs and define intermediate holding times (IMHTs). Three individual plates were prepared for 114 USP samples, four plates for 150 DSP samples, and three plates for 147 IMHT samples. Each plate included three plate controls, consisting of the same drug substance sample prepared in triplicate, to assess the inter-plate variability of the robotic sample preparation. The position of the three plate controls was defined so that the variability of the sample preparation was assessed at the start, intermediate point and end of the procedure. A thermally stressed sample (positive control) was also prepared and injected at the start, middle and end of each analytical sequence to confirm the detectability of mAb3 PQAs along each sequence. In total, 472 samples were prepared in ten 96-well plates and injected in ten analytical sequences using a single LC-MS instrument. All plate controls of each sample set (USP, DSP and IMHT) were injected together in the same analytical sequence to prevent sequence-to-sequence variability, if any. Any variability between plates was assessed with positive controls. Moreover, a predigested NIST mAb was injected as an SST to evaluate instrument performance and confirm system readiness.

**Figure 4:**
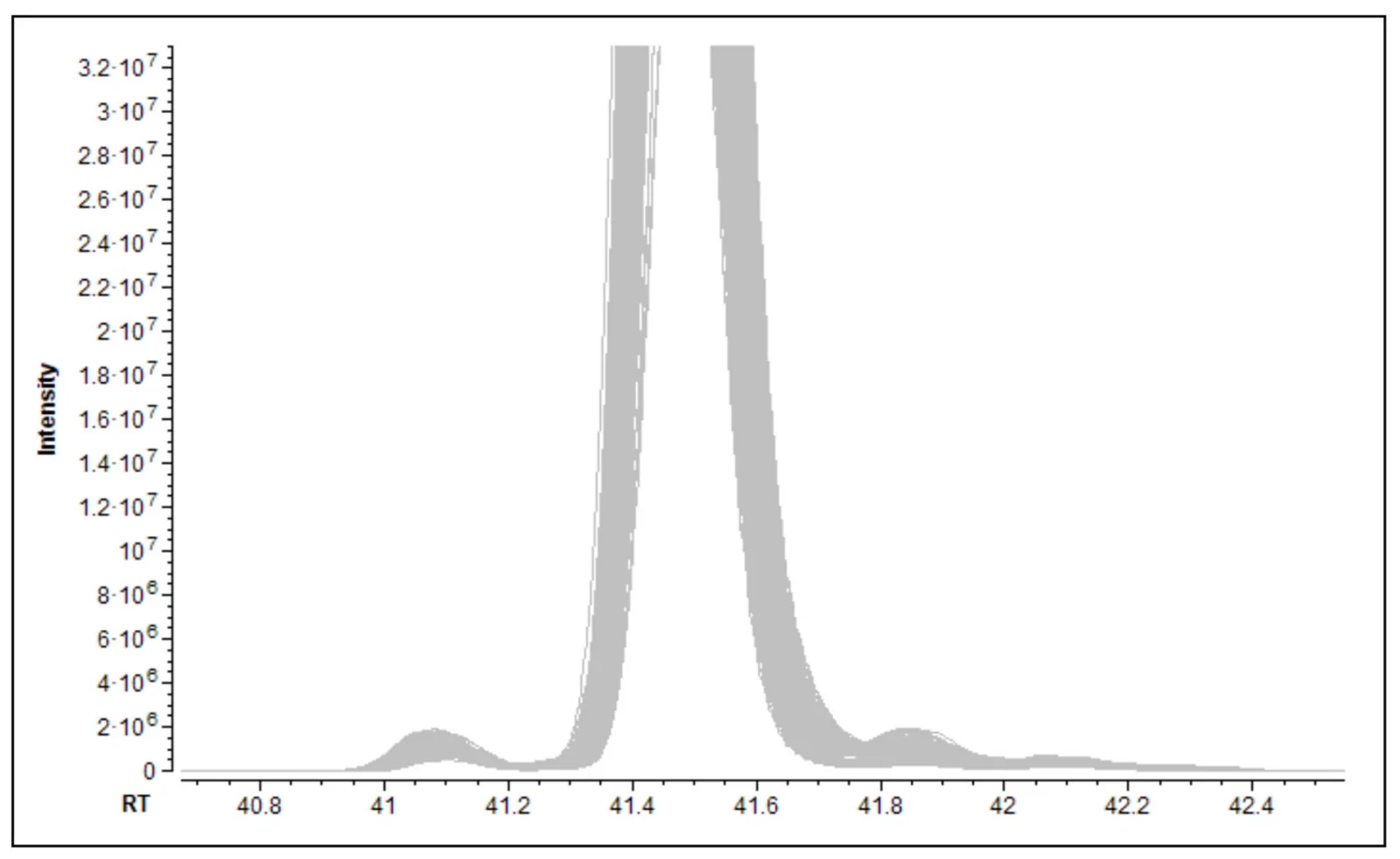
Overlay of 472 extracted ion chromatograms of the deamidated GFYPSDIAVEWESNGQPENNYK peptide after simultaneous data processing. The extracted ion chromatograms of the doubly charged deamidated precursor at *m*/*z* 1273.0613 together with the second isotope of the main native peptide is shown.

The 472 MS raw files were processed simultaneously to identify PQAs and to report their relative PQA abundance using the mAb3 project-specific MAM peptide library, which contained 86 unique PQAs. Figure 4 shows the extracted ion chromatograms of the deamidated GFYPSDIAVEWESNGQPENNYK peptide for the 472 samples after simultaneous data processing and chromatogram alignment, demonstrating the suitability of the MAM workflow to retain chromatographic separation across such a large sample set.

The mAb3 MAM library enabled the reporting of the relative abundance of asparagine deamidation, aspartate isomerization, succinimide, N-terminal pyroglutamate, C-terminal proline amidation, methionine and tryptophan oxidation, as well as 17 N-glycans including high-mannose and sialylated species. In addition, 25 glycated peptides and 14 truncated peptides were successfully monitored to assess mAb3 glycation and fragmentation status, respectively.

**Figure 5:**
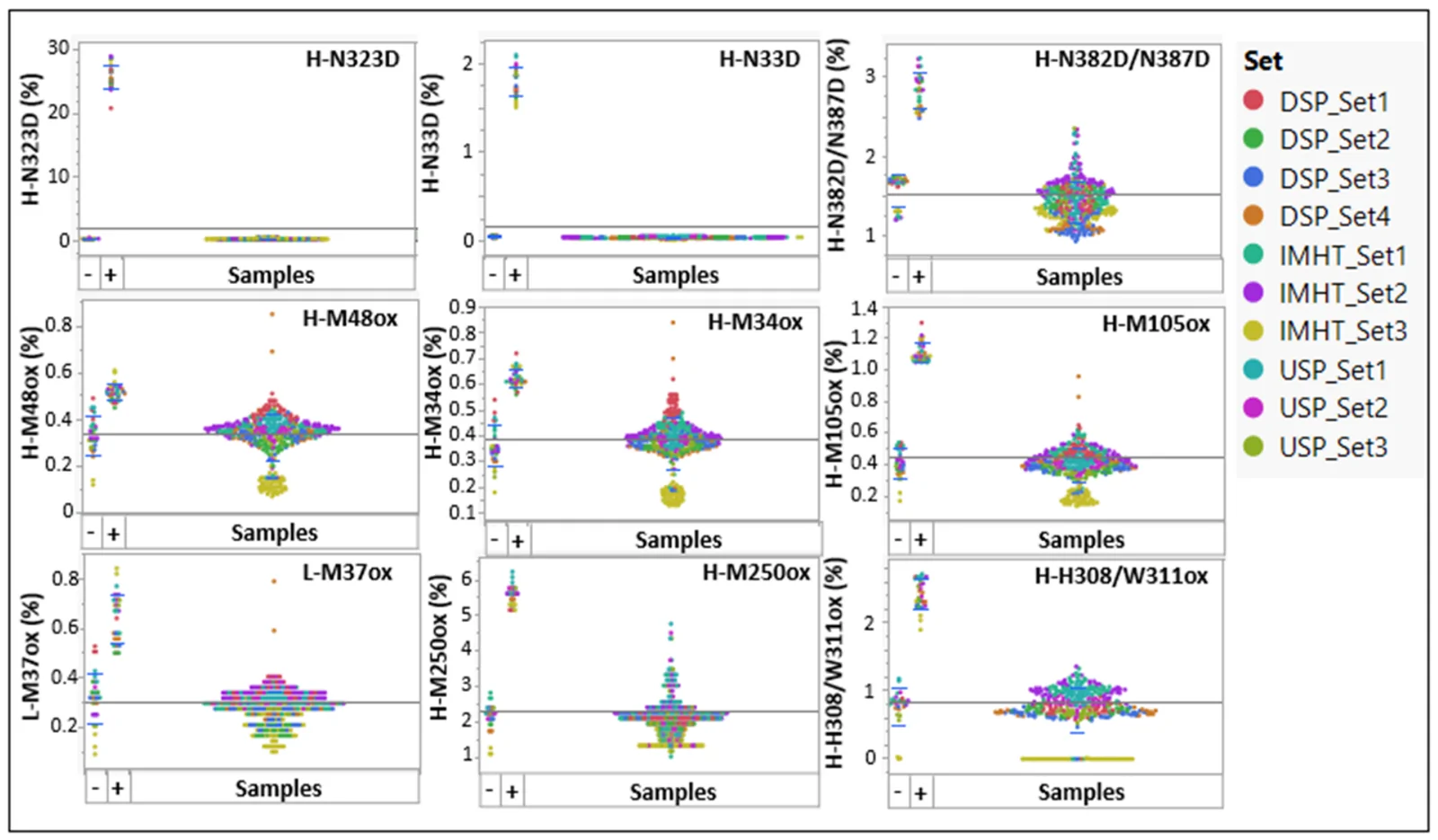
Monitoring and control of mAb3 deamidation and oxidation levels in process characterization samples. Plate controls’ (-), positive controls’ (+) and process characterization samples’ distributions are shown for deamidation and oxidation sites.

**Figure 6:**
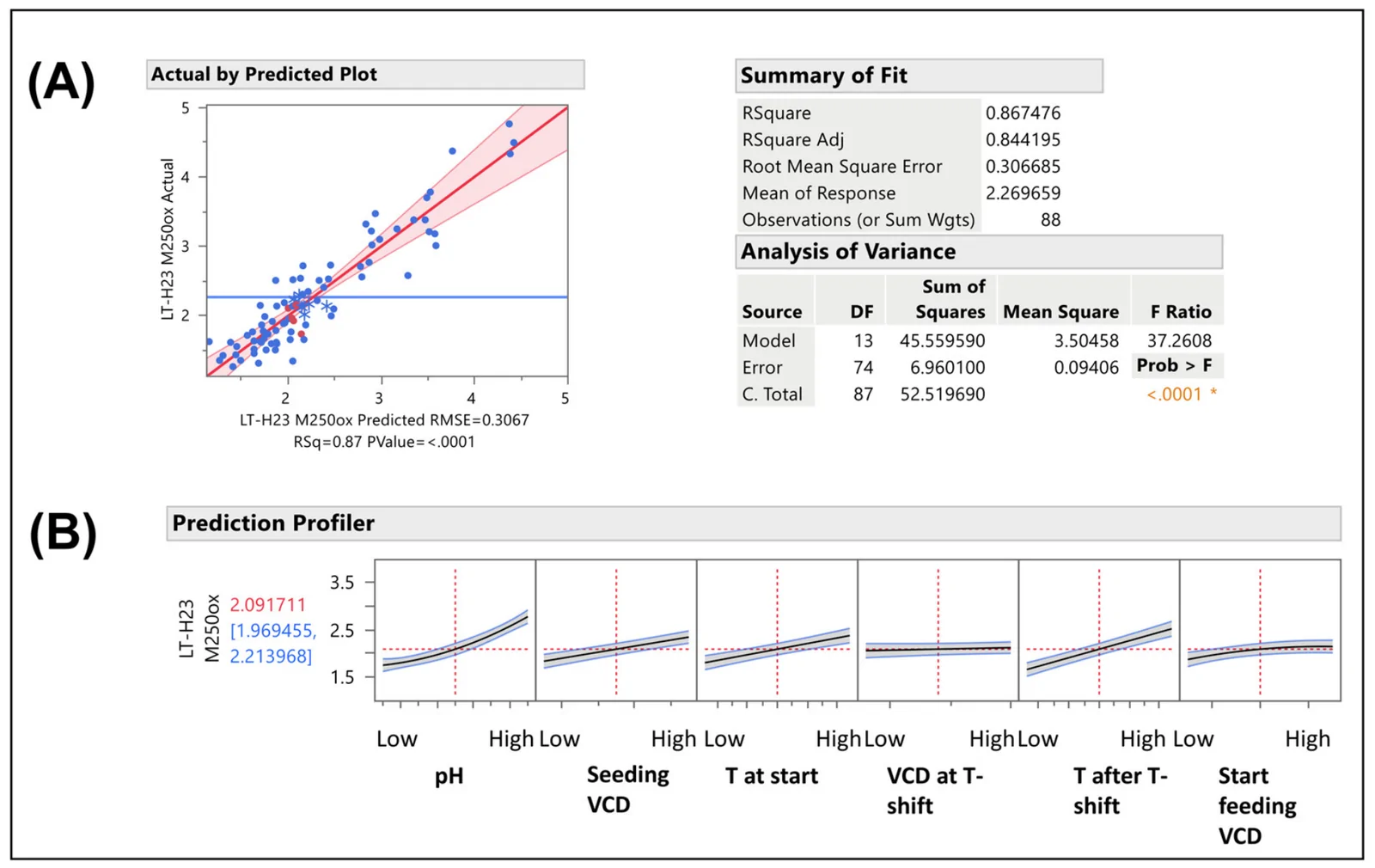
Influence of process parameters on mAb3 methionine 250 oxidation. (**A**) Actual-by-predicted plot and model fit of M250 oxidation in USP production bioreactor DoE samples. Red area depicts 95% confidence interval. * Significant model. (**B**) Prediction profiler plots for USP production bioreactor DoE indicating the influence of process parameters on M250 oxidation. Predicted values and 95% confidence intervals are shown.

After MS data evaluation and reporting, the overall impact of process parameter modulations on the relative abundance of mAb3 PQAs was assessed. Therefore, data from all PC studies were plotted together with the plate and positive controls (Figure 5). In general, mAb3 deamidation and oxidation levels remained consistently low across most PC samples, and within the range defined by the plate controls, confirming that these mAb3 attributes were not affected by individual unit operations of the DS process. Of note, the levels of oxidation for the IMHT sample set 3, including PC samples and plate controls, were about 0.2% lower than the other sample sets for most residues and up to 1% lower for M250ox and H308ox/W311ox, suggesting a minor plate effect for this IMHT sample set 3. However, oxidation at methionine residue 250 (M250) showed higher spread in PC samples compared to plate controls, indicating an impact of the manufacturing process. Accordingly, this response was further investigated. Figure 6 shows the DoE modeling results for the oxidation at M250 of mAb3 for the USP process parameters, indicating a good correlation with the experimental data. After reduction to significant model terms, the model contains in total 13 terms with 6 main effects, 5 interaction effects and 2 quadratic effects, while the term “VCD at T-shift” was kept in the model in accordance with the effect hierarchy principle. The process parameters with the largest impact on M250 oxidation were “temperature after temperature shift” and “pH of the culture medium”. These findings from the DS PC study also corroborated the results from the MAM sample preparation robustness study, which showed a relationship between increased oxidation levels with higher denaturation pH and digestion pH. This example showcases the power of this MAM platform which enabled high-resolution process characterization with a large number of samples to clearly identify individual process parameters and their impact on the CQA M250 oxidation.

**Figure 7:**
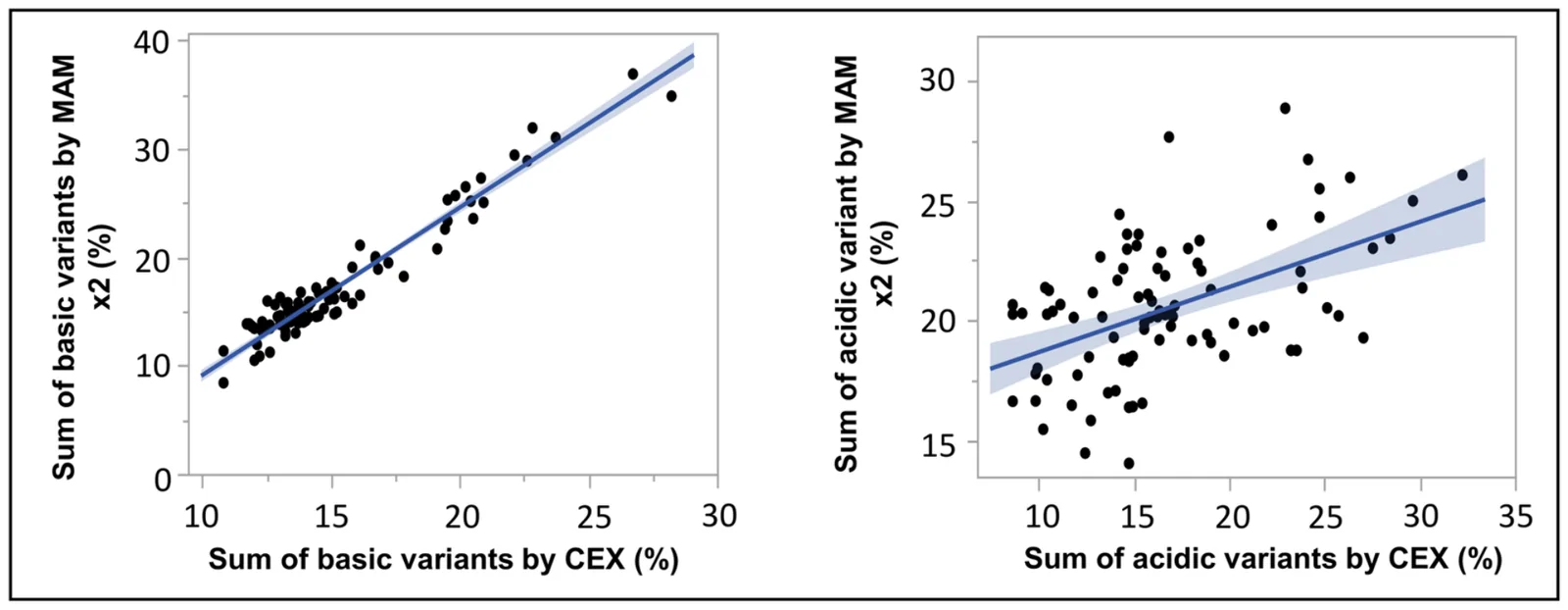
Correlation of mAb3 charge variants determined by MAM and CEX-HPLC for USP samples. Levels of individual heavy chain basic variants N-terminal glutamine, C-terminal glycine loss and amidation and succinimide at D278 were summed with a correction factor of 2 against the sum of basic variants by CEX. Blue area represents 95% confidence intervals.

Charge variant analysis can bring valuable insights into the impact of process parameters on charged PQAs, such as sialic acids, deamidation, C-terminal extension, etc. While CEX measures the levels of charge variants for an intact molecule, i.e., bearing two heavy and two lights chains, MAM determines the PQA abundance at peptide level without considering combinatorial distribution. At low PQA abundance, a correction factor of 2 can be used for the comparison of MAM and CEX data [27]. Figure 7 shows the correlation of the sum of charge variants by MAM compared to the sum of charge variants determined by CEX-HPLC for USP samples. MAb3 basic charge variants were characterized as heavy chain N-terminal glutamine, C-terminal glycine loss and amidation and succinimide at D278 by CEX-HPLC peak fractionation and LC-MS/MS analysis. A strong correlation was obtained between the sum of basic variants by CEX and by MAM, most particularly due to the presence of significant heavy chain N-terminal glutamine variant levels. However, the behavior of individual charge variants may differ for individual process parameters as shown below. Conversely, the correlation between MAM and CEX data was poor for acidic variants. This poor correlation for acidic variants may be multifactorial due to the impact of (1) structural level, (2) quantitation bias, and (3) process on individual PQAs: (1) MAM monitors a discrete number of site-specific variants at the peptide level while CEX assesses a global charge distribution at the intact level; (2) the estimation of variants may be inaccurate at the peptide level due to differences in ionization properties and at the intact level due to the co-elution of a combination of variants; (3) the correlation may therefore be impaired in cases where the process parameters have different impacts across acidic variants as estimated by CEX and MAM. MAb3 acidic charge variants monitored by MAM included sialylated N-glycans (FA2G1S1, FA2G2S1, and FA2G2S2), deamidation at heavy chain N33, N295, N323, N382, N387 and isomerization at light chain D171, as well as light chain N-terminal glycation and heavy chain CDR 3 glycation at K97, which were selectively enriched in the acidic CEX fraction during characterization. Although we previously characterized glycation as an acidic variant [15], most glycation sites co-elute with the main CEX peak and do not have an impact on the apparent charge. Finally, an offset may still exist which may be due to the difficulty in estimating glycation at the peptide level. The relative abundances of the glycated peptides are provided as an estimate and may be inaccurate as the ionization properties of glycated peptides are expected to vary substantially due to the difference in size and charge as compared to shorter fully cleaved peptides. Intact RP-MS analysis after N-deglycosylation estimated that approximately 19% of mAb3 is glycated.

**Figure 8:**
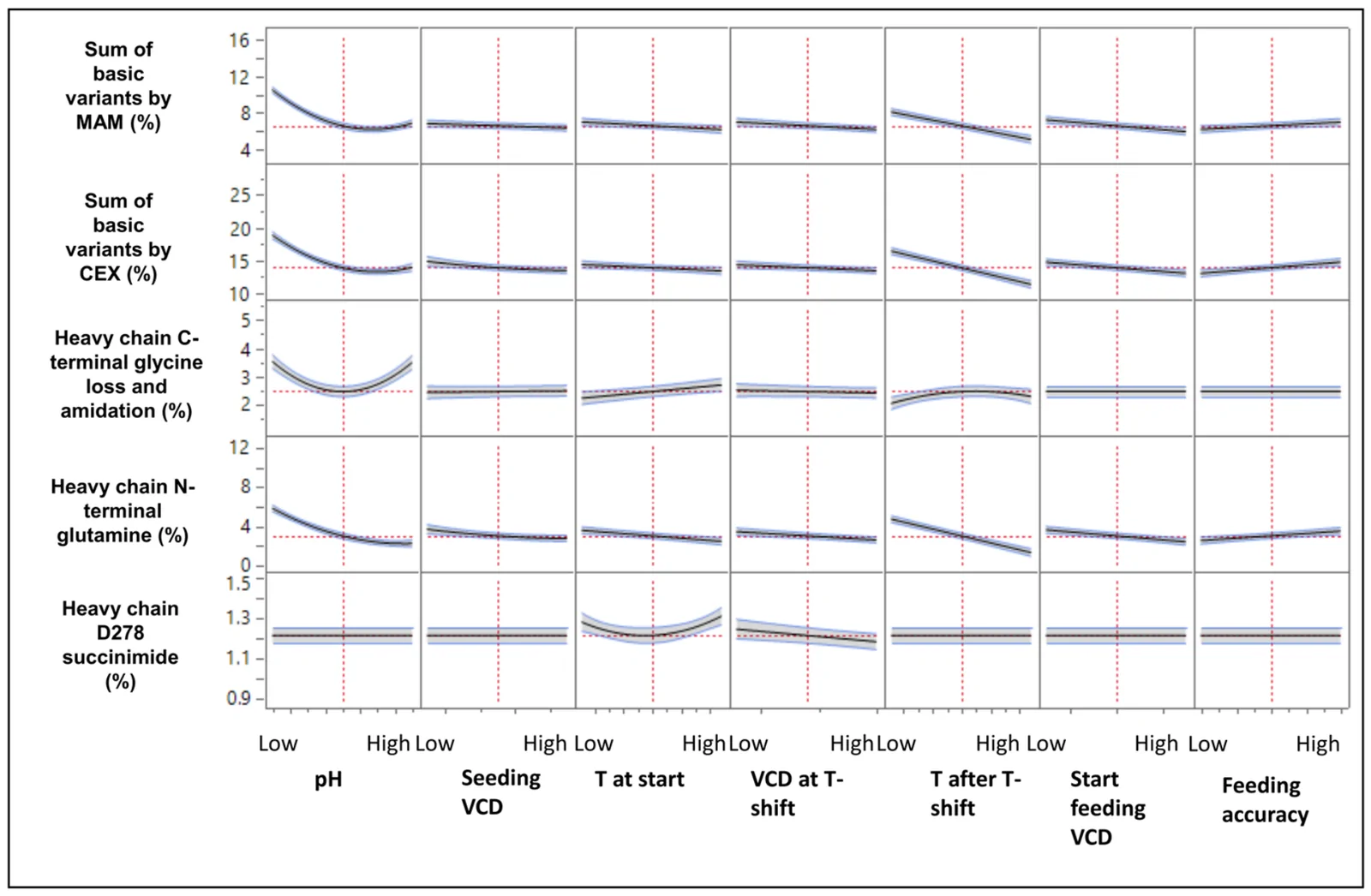
Influence of process parameters on mAb3 basic variants assessed by MAM and CEX. USP process parameters are shown with their respective prediction profiler plots for basic variants of heavy chain C-terminal glycine loss and proline amidation, heavy chain N-terminal glutamine and succinimide in the heavy chain constant region, as well as for the sum of basic variants by MAM and CEX. Prediction trace and error bars show predicted values and 95% confidence intervals.

Figure 8 shows the behavior of mAb3 basic variants within the USP process parameter ranges. Based on the established correlation between MAM and the conventional method CEX-HPLC, the same conclusions in terms of the impact process parameters on the sum of basic variants could be drawn. However, only MAM provided additional insights into the impact of process parameters on individual PQAs. At the individual PQA level, the pH of the cell culture had the strongest impact on the heavy chain N- and C-terminal variants as compared to the succinimide. Notably, the levels of the C-terminal glycine loss and proline amidation were the lowest at the center point pH, which may be linked to the pH optima of the enzymes carboxypeptidase B and peptidylglycine alpha-hydroxylating monooxygenase, respectively. The temperature of the main stage bioreactor, in particular after the temperature shift, had an inverse relationship with the level of N-terminal glutamine. Succinimide formation was only influenced to a lesser extent by process parameters such as the temperature at start and viable cell density at the temperature shift. Figure 8 illustrates an example where the conventional method CEX-HPLC would potentially lead to incorrect conclusions, since the individual basic variants monitored by MAM do not exhibit the same dependencies on process parameters as the sum of basic variants by CEX-HPLC.

**Figure 9:**
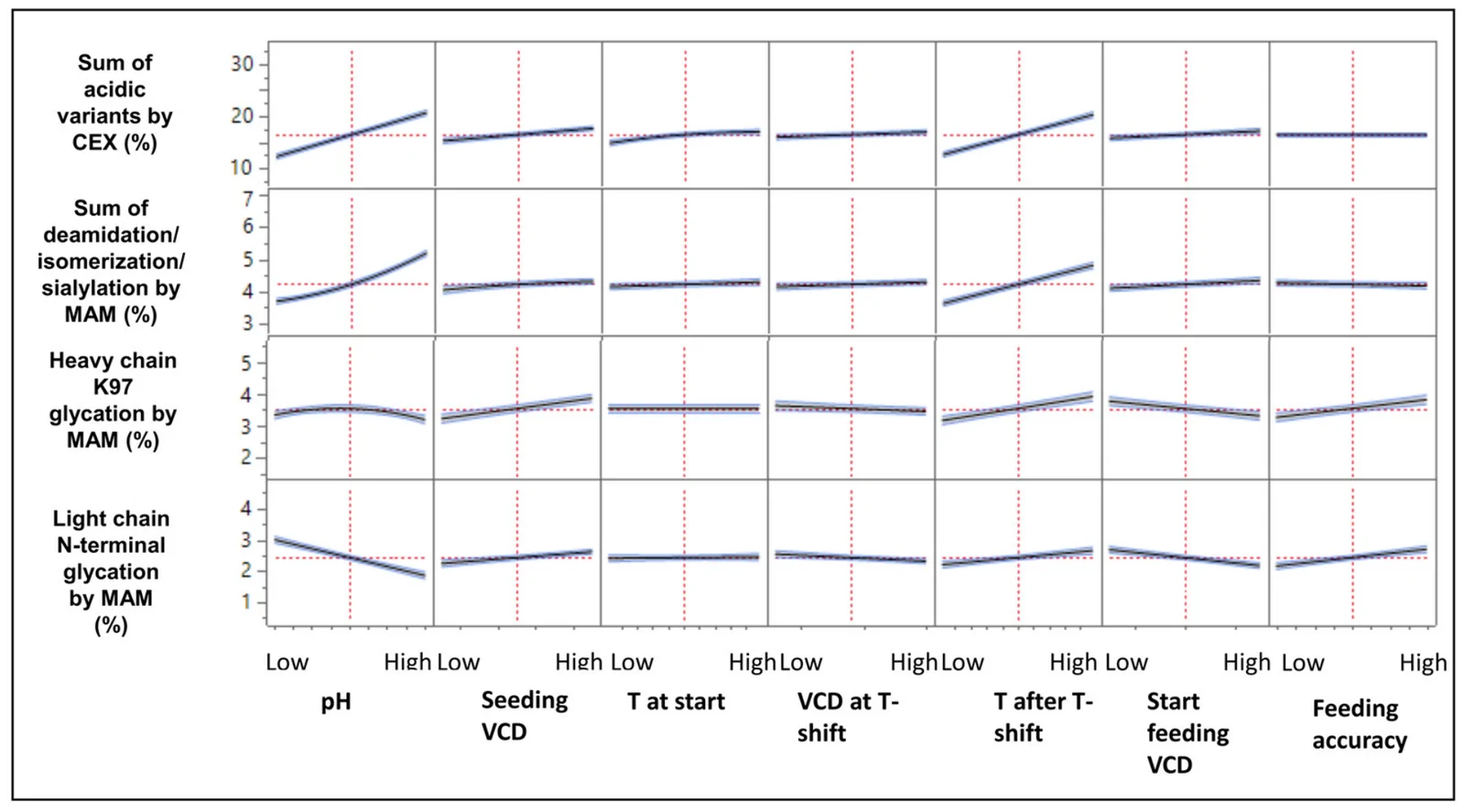
Influence of process parameters on mAb3 acidic variants assessed by MAM and CEX. USP process parameters are shown with their respective prediction profiler plots for the sum of acidic variants by CEX and sum of deamidation/isomerization and sialylation by MAM, heavy chain glycation at K97 and light N-terminal glycation. Prediction trace and error bars show predicted values and 95% confidence intervals.

Figure 9 displays the influence of process parameters such as viable cell density, temperature and feeding accuracy on acidic variants determined by CEX, and those monitored by MAM grouped as the sum of deamidation/isomerization/sialylation, glycation in heavy chain CDR3 and light chain N-terminal glycation. The DoE models for the sum of deamidation/isomerization/sialylation by MAM and acidic variants by CEX indicate a very comparable behavior for all tested process parameters. Prediction profiler plots of the monitored glycation sites also show a strong impact of temperature on this attribute. However, the prediction profiler plots of glycation differed from those of deamidation/isomerization/sialylation, especially for the impact of pH. The basic pH in the bioreactor had a strong impact on deamidation at N382 and N387. Of note, deamidation at N323 remained low in the PC samples, while it reached approximately 25% in thermal stress positive control samples exposed at 40 °C for 3 months. The two glycation sites had slightly different behavior with the pH of the bioreactor. These results show that different acidic variants may exhibit different behaviors during PC, even from the same class of attribute, and that monitoring the sum of charge variants with conventional methods may remain limited in building process understanding. These observations suggest that MAM can enable site-specific glycation monitoring, where the sum of acidic variants by CEX is the result of different dynamics of co-eluting variants. Relying on conventional methods for the monitoring of acidic variants may lead to incorrect process conclusions or “optimization” as changes in process parameters like pH and temperature may result in different co-eluting acidic variants. These findings show that correlation between MAM and conventional methods can be impaired when using PC samples generated from multiple process parameters, impacting different co-eluting variants.

**Figure 10:**
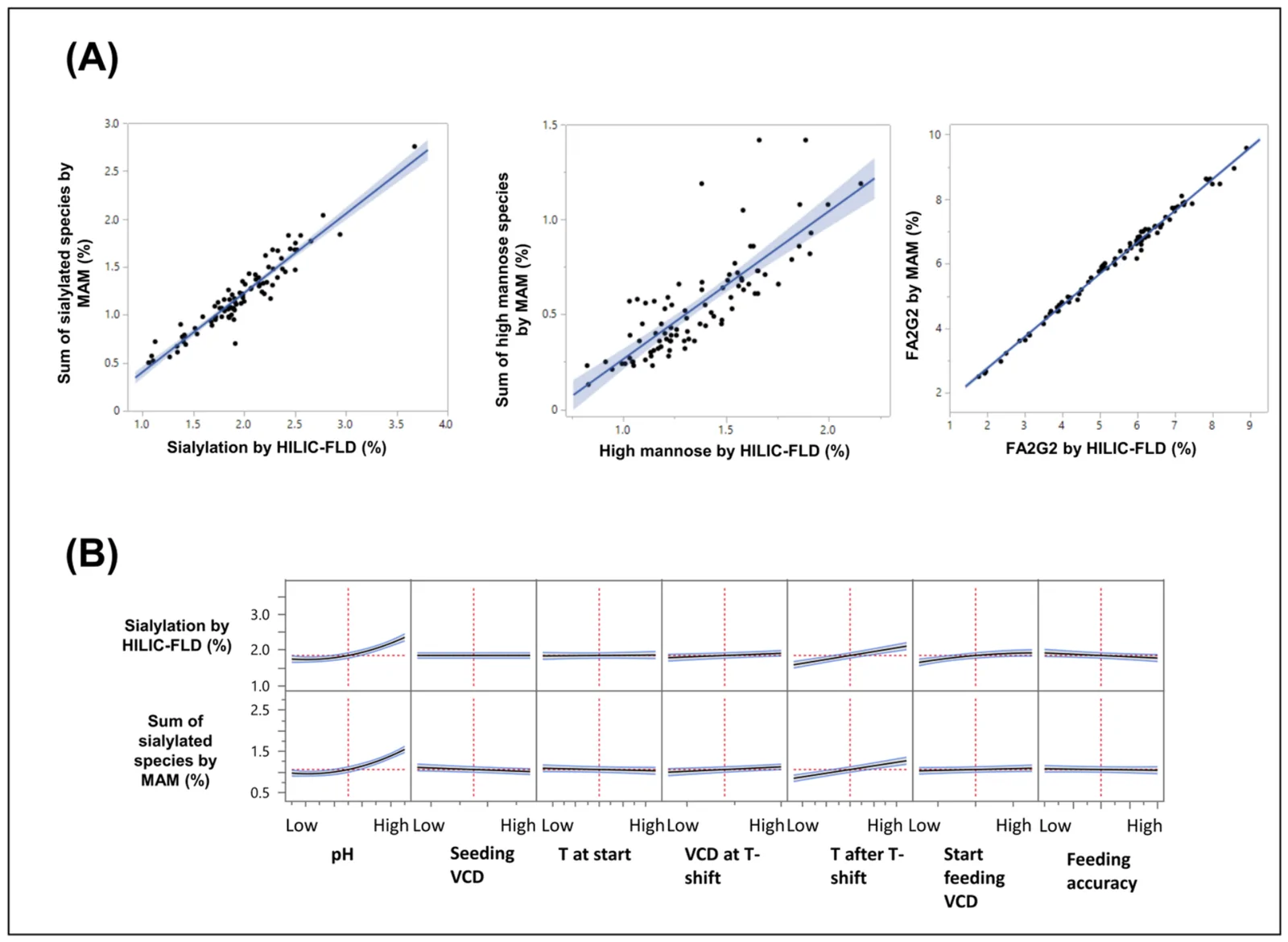
Monitoring N-glycans by MAM and HILIC-FLD. (**A**) Correlation between MAM and N-glycan profiling by HILIC-FLD for high-mannose M5, M6, M7 and M8, sialylated FA2G1S1, FA2G2S1, FA2G2S2 and galactosylated FA2G2 N-glycan. Blue area represents 95% confidence intervals. (**B**) USP process parameter prediction plots for the sum of sialylated species FA2G1S1, FA2G2S1, and FA2G2S2 by MAM and sialylation by HILIC-FLD. Prediction trace and error bars show predicted values and 95% confidence intervals.

Another important CQA is the type of N-glycan attached to the protein backbone in the Fc domain of mAbs if effector functions are contributing to their mode of action [28]. N-glycosylation is a PTM which may be impacted by, e.g., the pH of cell culture and dissolved oxygen tension during the manufacturing process [29]. N-glycan profiling is typically monitored by HILIC-FLD after N-glycan release by, e.g., PNGase F, and labeling with a fluorophore. In this study, the mAb3 N-glycosylation profile and site occupancy of >99% was retrieved by MAM. Figure 10 shows the correlation between MAM and N-glycan profiling for high-mannose, galactosylated FA2G2 and sialylated species including FA2G1S1, FA2G2S1, and FA2G2S2. The correlation between MAM and HILIC-FLD was good for sialylation and galactosylation. However, higher levels of high-mannose species were monitored by N-glycan profiling compared to MAM, while the upper end of the correlation revealed outliers by MAM for four USP samples. These four samples exhibited higher levels of M6, and to a lesser extent of M7 and M8 N-glycans, compared to other samples as determined by MAM. Notably, HILIC-FLD peak characterization by LC-MS/MS confirmed the co-elution of M6 with other species such as bisecting biantennary galactosylated species FA2BG1 or triantennary species FA3G1. MS/MS spectra were lacking sufficient diagnostic ions to distinguish FA2BG1 from FA3G1. Due to its higher specificity, MAM was able to resolve and track the behavior of N-glycan species co-eluting in HILIC under this experimental setup and therefore provided greater resolution than the conventional method during PC. Among the USP process parameters studied, the pH and temperature in the bioreactor had the most impact on the levels of sialylation as shown in Figure 10B.

**Figure 11:**
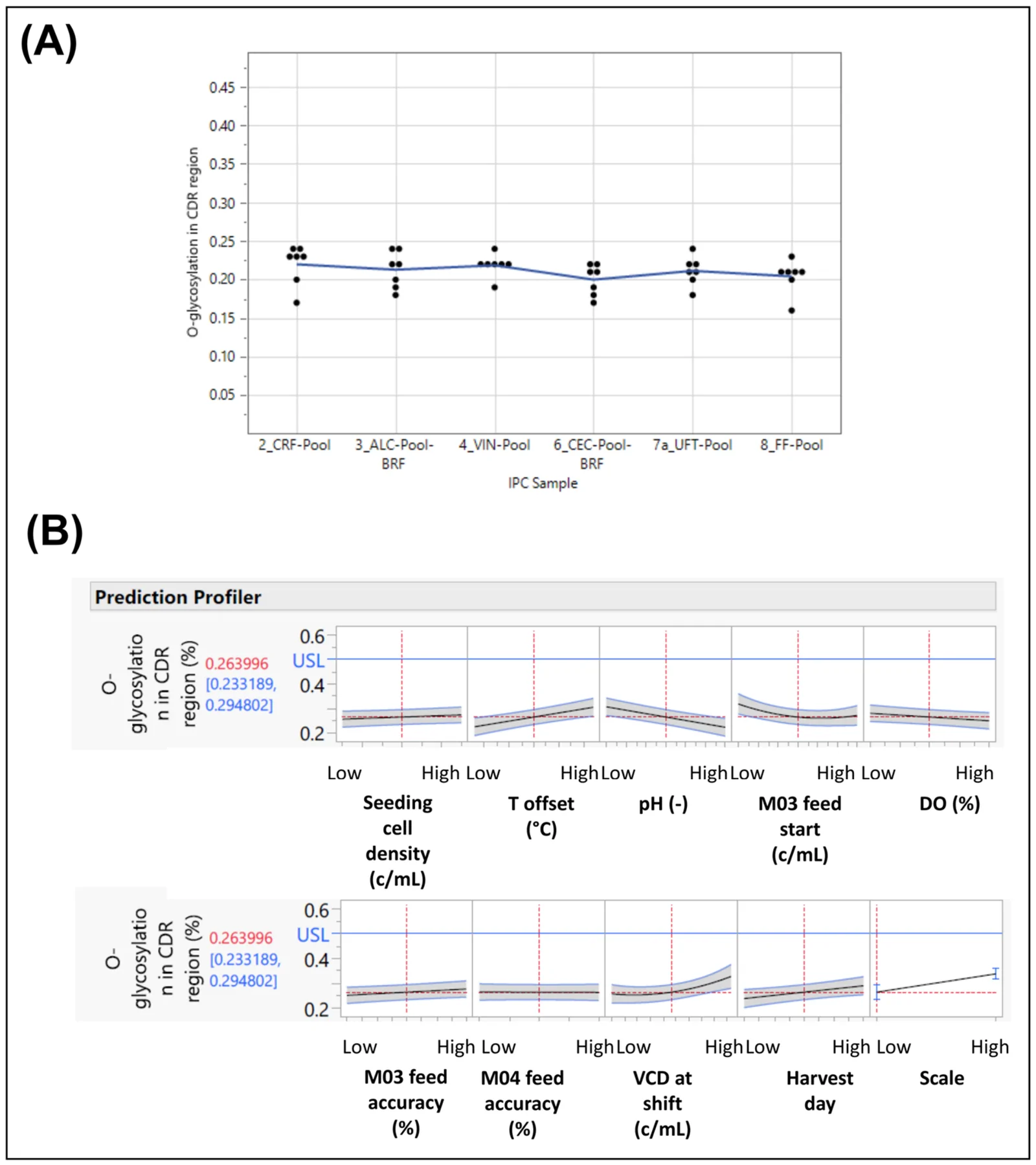
Monitoring O-glycosylation in mAb2 CDR. (**A**) Level of O-glycosylation along the different purification steps. (**B**) USP process parameters of O-glycosylation in mAb2 CDR. Prediction trace and error bars show predicted values and 95% confidence intervals.

Beyond the PQAs most commonly monitored for IgGs, O-glycosylation has been reported in various immunoglobulins, and recently identified in the constant region of an IgG4 at levels below 0.1% [30]. We show that the MAM platform provides enough sensitivity to monitor low levels of O-glycosylation in the CDR of mAb2 ranging from 0.2 to 0.3% in the purification steps (Figure 11) and to identify process parameters with an impact on this CQA like the temperature offset or the viable cell density at the temperature shift.

## 3. Discussion

This study highlighted the benefits of applying MAM for PC, as MAM revealed the impact of process parameters on individual site-specific CQAs. Thus, MAM may help to refine parameter setpoints and acceptable ranges with higher specificity than with conventional methods, for which indirect readouts, e.g., “the sum of variants”, may be insufficient or lead to incorrect conclusions. In addition, identifying process parameters that impact only less critical PQAs may allow setting wider process parameter ranges and thus enable more flexibility for process adaptions during product lifecycle management. Furthermore, demonstration of consistent CQA levels across multiple CPPs may support justification of a robust overall control strategy even in the absence of confirmatory release testing. These findings were possible due to the implementation of a robust MAM platform, demonstrated by ensuring the specificity, linearity and reproducibility of measurements across six laboratories even for labile modifications such as succinimide, deamidation, isomerization and oxidation. By applying this MAM platform to the support of PC studies, we confirm that the MAM platform performance can be maintained for the analysis of more than 600 samples. By leveraging an automated, robust and harmonized MAM platform across multiple geographies, we scaled up and streamlined the MAM capabilities to enable fast data analysis of large project-specific PQA libraries along the development phases. Beyond the automation of sample preparation, this MAM platform addressed limitations in sequential data analysis by leveraging parallel data processing from multiple samples and analytical sequences. This MAM platform allowed us to report up to 100 individual PQA abundances from 472 samples and controls acquired with one instrument, and more than 600 samples and controls acquired on two instruments within one week. This throughput was enabled by simultaneous and consistent PQA peak boundary integration across samples. The risk of not detecting individual retention shifts by handling long analytical sequences with multiple instruments were addressed by automated online library recalibration and validation, enabling analysis and review by non-MS experts. This report shows how MAM can be applied to build process understanding by monitoring CQAs in studies with a large sample number such as a PC study while addressing the software limitations to enable the processing and comparison of up to a hundred PQAs.

This performance can be leveraged to enable consistent CQA monitoring with sustained performance during the development of new biopharmaceutical products across multiple development sites in a global pharmaceutical organization. Furthermore, limitations related to method artefactual deamidation and oxidation were addressed. While oxidation can be maintained at low levels using TFA as compared to formic acid, and methionine in sample preparation [10], we demonstrated that both artefactual methionine oxidation and isomerization/deamidation can be maintained at low levels even for the analysis of large sample sets. This report also shows that the manufacturer and quality of the TFA stock solution used for mobile phase preparation are key method parameters to be tracked to ensure the performance of MAM in a global setup. We hypothesized that the site-specific oxidation induced by TFA may be driven by the presence of impurities such as metals [24,26]. Adding EDTA as proposed by other groups may help to control the levels of oxidation by trace metals [31], while other have shown that the use of chelating agents like EDTA or DTPA may accelerate the leaching of metal ions from equipment [25,32]. The control of trace levels of metals in reagents may be an alternative solution to prevent unexpected method-induced oxidation [33].

In mAb3, controlling artefactual oxidation was pivotal to revealing the unexpected impact of USP parameters on oxidation at M250, a CQA that may impact PK/PD at high oxidation levels [34]. The MAM results correlated well with the sum of charge variants determined by CEX and N-glycan mapping by HILIC-FLD. The correlation between MAM and N-glycan mapping was also demonstrated for low-level sialylated species and for galactosylation. However, an offset may exist between CEX and MAM results, as the estimation of glycation by MAM depends on the number of glycation sites considered, the ionization properties of the missed cleaved peptide bearing the glycation and the reference peptides used for calculations. An offset between MAM and CEX data can be corrected by considering the two sets of chains in mAbs [27], but such correlations can be inaccurate when variants are not well separated from the main or distributed across multiple peaks in CEX. A previous study has focused on the prediction of charge variants with MAM data using multivariate data analysis and calibration models, which may be considered to correct such offsets in estimations and predictions [18]. The relevance of such correlations relies on the accuracy of both methods to monitor individual variants. However, the identification of CEX peaks typically requires fractionation from unstressed and stressed materials and subsequent characterization of isolated fractions by complementary techniques. It is likely that variants not enriched during fractionation using the stressed material may co-exist at lower levels, while being highly abundant at the USP process parameter settings studied during a PC exercise. A challenge for the implementation of MAM in place of conventional techniques is the prerequisite to correlate highly specific MAM results with indirect readouts obtained with conventional methods. Correlation of MAM and CEX results may be easily obtained for stressed samples, but such a correlation may be more challenging for samples from USP unit operations or when the estimation of a modification, e.g., glycation, may be impaired by different ionization efficiencies of the peptides used for the monitoring.

This study demonstrated that MAM has superior capabilities compared to the CEX method in monitoring relevant CQAs such as deamidation, isomerization, glycation and O-glycosylation in the CDR of mAbs. Glycation at multiple residues could not be monitored via the sum of acidic variants by CEX, as a population of glycated mAb3 molecules co-eluted with the CEX main peak and only exposed glycation sites in the CDR and N-terminal to the light chain were separated by CEX. We also show that monitoring the sum of charge variants by CEX lacks specificity to study the impact of individual process parameters on individual CQAs. MAb3 acidic variants such as glycation in CDR and heavy chain deamidation, as well as basic variants such as succinimide formation and C-terminal proline amidation, showed different behaviors upon process parameter variation. Therefore, relevant changes to CQAs may be masked by non-relevant changes in less critical PQAs using CEX. These examples showed that the sum of charge variants as the classical method of choice to evaluate the underlying PQAs could potentially lead to incorrect conclusions, since the different charge variants did not exhibit the same dependencies on process parameters. A worst-case scenario may be that a process parameter is not considered as critical from studying the sum of basic/acidic variants, while monitoring a specific CQA (among the basic/acidic variants) may lead to the classification of the parameter as being critical. The site-specific MAM enables high-resolution process characterization by monitoring the different variant responses to individual process parameters, thus enabling the identification of CPPs with more specificity and helping to define more appropriate acceptable ranges for process parameters in manufacturing. Several USP CPPs and their impact were identified on acidic variants including deamidation, isomerization and glycation, as well as basic variants, N-glycosylation, clipping, and O-glycosylation in the CDR. Additionally, such in-depth product and process understanding can be leveraged to predict the impact of planned process changes or unwanted deviations during routine manufacturing on product quality, thus facilitating lifecycle management and investigations to ensure consistent manufacturing of a safe and efficacious product. Overall, MAM is expected to significantly increase our product and process understanding and may lead to the optimization and fine-tuning of the manufacturing process control strategy, i.e., by definition of acceptable ranges or in-process controls, for modifications such as O-glycosylation that cannot be monitored by conventional methods typically applied for the quality control of biopharmaceutical products.

## 4. Materials and Methods

### 4.1. Samples

Monoclonal antibodies 1, 2 and 3 (mAb1, mAb3 and mAb2) were manufactured at Novartis Pharma AG (Basel, Switzerland). Control and stressed samples were used to perform characterization work and build the project-specific MAM peptide library using relevant pH, thermal and oxidative stress conditions. For the MAM platform qualification with mAb1, oxidative stress material was spiked at various levels in unstressed material. For the qualification of isomerization and succinimide levels, thermal stress material was spiked at various levels in unstressed material, while basic pH stress was used for the qualification of deamidated residues.

For mAb1, the oxidation linearity was assessed by spiking mAb1 control samples with an oxidized mAb1 sample. The linearity samples were produced in one coordinating laboratory and distributed to the five other laboratories.

For mAb2, 317 USP and 242 DSP PC samples were obtained. A thermal stress sample was used as a positive control for PQA detection, and an unstressed reference material was used as a plate control.

For mAb3, 114 USP and 150 DSP process characterization samples were derived from in-process control samples of a mAb3 manufacturing process. Additionally, 147 IMHT samples were collected. A thermal stress sample was used as a positive control for PQA detection, and an unstressed drug substance was used as a plate control.

### 4.2. Reagents

Lys-C (MS grade, 125-05061) was purchased from Wako (Osaka, Japan), modified porcine Trypsin (MS grade, V5280) was purchased from Promega (Madison, WI, USA), 8M Guanidine hydrochloride (24115) was purchased from Pierce (Rockford, IL, USA), EDTA disodium salt, dehydrate (E5134-250G), was purchased from Sigma (St. Louis, MO, USA), 1M Tris-hydrochloride pH8 solution (Ultrapure, 15568-025) was purchased from Gibco (Paisley, UK), Calcium chloride dihydrate (CaCl2) was purchased from VWR (Solon, OH, USA), dithiothreitol (43815-1G) and iodoacetamide (Bioultra, I1149-5G) were purchased from Sigma (St. Louis, MO, USA), acetonitrile (LC-MS grade, 9821) was purchased from Biosolve (Valkenswaard, Netherlands), water (LC-MS grade, 9823-02) was purchased from J.T.Baker (St. Louis, MO, USA), Isopropanol (LC-MS grade, 34965) were purchased from Fluka (St. Louis, MO, USA), trifluoroacetic acid (TFA) ampoules were purchased from Pierce (Rockford, IL, USA, MS grade, 28904) and Sigma-Aldrich (St. Louis, MO, USA, HPLC grade, 91707), and TFA bottles (LC-MS grade, 84868.180) were purchased from VWR (Heverlee, Belgium).

### 4.3. Automated Sample Preparation for Characterization

Control samples and stressed material were used for the characterization of mAb1, mAb2 and mAb3. The robotic sample preparation and Lys-C/Trypsin digestion was performed using a STARLET robotic system (Hamilton, Bonaduz, Switzerland) equipped with a TRobot II PCR thermal cycler (Biometra, Jena, Germany). Briefly, the sample protein concentration was normalized to 10 mg/mL in a 96-well PCR plate (Eppendorf, Hamburg, Germany). Protein disulfide bonds were reduced for 1 h at 37 °C using 5 mM DTT under denaturing and slightly alkaline solution conditions (4.7 M Gnd-HCl and 38 mM Tris-HCl, pH 8.0). Then, alkylation of reduced cysteines was performed for 1 h in the dark with 9 mM IAM. DTT was used to quench the alkylation. The samples were diluted in 25 mM His-HCl buffer, pH 6.0, before combined Lys-C/Trypsin digestion at 37 °C for 20 h with a 1:50 (*w*/*w*) enzyme-to-protein ratio. TFA at a final concentration of 0.1% (*v*/*v*) was used to stop the enzymatic reaction.

### 4.4. LC-MS/MS Data Acquisition for Characterization

An aliquot of 100 µL (22 µg) of the digested samples was injected on a Waters Acquity UPLC Peptide HSS T3 column (100 Å pore size, 1.8 µm particle size, and 2.1 × 150 mm, Milford, MA, USA) at 0.2 mL/min and 40 °C using a Thermo Vanquish Horizon UHPLC system coupled to a Thermo Orbitrap Fusion Lumos mass spectrometer (Thermo Fisher Scientific, Reinach, Switzerland) via an electrospray ionization source. Peptide separation was performed with a 69 min linear gradient from 10 to 47.5% B (0.1% TFA in ACN) after a 5 min desalting step at 100% A (0.1% TFA in mQ water) and a 1 min % B increase from 0 to 10% B. The column was then washed for re-equilibration for 34 min. The instrument was operating in positive ion mode with a capillary voltage of 3.8 kV, ion transfer tube temperature at 275 °C, sheath gas flow rate at 35, and auxiliary gas flow rate at 10. MS data were acquired with a data-dependent acquisition method with a 3 s duty cycle using Xcalibur software version 4.1.5 (Thermo). Full MS scans were recorded at 120,000 resolution (standard AGC target, 60% RF lens, 50 ms maximum injection time, and 300–2000 *m*/*z* scan range). Precursor ions were isolated with a quadrupole (1.6 *m*/*z* window) before fragmentation by collision-induced dissociation (CID, collision energy: 30, activation time: 10, activation Q: 0.25, and 500 ms maximum injection time) and higher-energy collisional dissociation (HCD, collision energy: 33.25–36.75 and automatic maximum injection time selection) and MS/MS data acquisition at 30,000 resolution.

### 4.5. LC-MS/MS Data Analysis for MAM Peptide Library Building

The characterization work was performed using an unbiased differential and statistical data analysis approach in Genedata Expressionist^®^ Refiner MS and Analyst v13.5 to 2025.1 (Genedata, Basel, Switzerland), as previously described [15,16].

Briefly, data preprocessing including RT alignment, chromatogram smoothing, noise subtraction, *m*/*z* peak detection, and isotope clustering were performed before peptide mapping. Processed data were searched against the expected respective mAb1, mAb2 and mAb3 protein sequences with cysteine carbamidomethylation as fixed modification, and consensus and non-consensus N/O-glycans and degradation, e.g., methionine/tryptophan oxidation, glycation, succinimide, deamidation were set as variable modifications. Clippings were also retrieved with semi-specific searches. Mass accuracy was better than 20.0 ppm, and MS/MS spectra were manually confirmed. Finally, the RT, mass, and *m*/*z* values of relevant peptides were compiled into a project-specific MAM peptide library.

### 4.6. Automated Sample Preparation for LC-MS MAM Analysis

For MAM analysis of mAb1, mAb2 and mAb3, the concentration of each protein sample was normalized to 2.5 mg/mL using the STARLET robotic system (Hamilton) and the same procedure conditions described above for characterization.

The mAb1 linearity samples prepared by the coordinating laboratory were digested independently by the six participating laboratories using the same automated sample preparation, robotic script and Hamilton robot for five laboratories. One laboratory performed the automated sample preparation with a Freedom EVO 150 robot (Tecan, Männedorf, Switzerland) equipped with a TRobot II PCR thermal cycler (Biometra, Jena, Germany).

The mAb2 MAM automated sample preparation was performed with two Hamilton robots using the same automation script. Laboratory 1 processed 242 DSP samples in 12 plates and laboratory 2 processed 317 USP samples in 13 plates. Two replicates of the reference material were prepared for each plate. The positive control was prepared in a single plate and aliquoted before injection for each analytical sequence corresponding to each DSP/USP plate.

For mAb3, 472 samples including controls were prepared in ten 96-well PCR plates using a single Hamilton robot, distributed in three plates for the 114 USP samples, four plates for the 150 DSP samples and three plates for the 147 IMHT samples. Each plate contained one plate control (one unstressed sample) in triplicate and one positive control (thermal stress sample) in triplicate, for a total of thirty plate controls and thirty-one positive controls.

### 4.7. LC-MS MAM Data Acquisition

The mAb1 linearity samples were injected on Thermo Vanquish Horizon UHPLC coupled to orbitrap analyzers in all six laboratories. Three laboratories used a Thermo Exactive Plus mass spectrometer with a heated electrospray ionization source piloted with Chromeleon 7.2 (Thermo, Reinach, Switzerland). The three other laboratories used a Thermo Q-Exactive mass spectrometer with a heated electrospray ionization source piloted with Xcalibur (Thermo).

MAM data acquisition of mAb3 samples was performed with a single Thermo Vanquish UHPLC system coupled to a Thermo Exactive Plus mass spectrometer with a heated electrospray ionization source. The LC-MS system was piloted with Chromeleon 7.2 (Thermo). About 4 µg of protein digest was injected for each mAb1, mAb3, and mAb2 sample. The same chromatographic conditions described above for characterization were used to streamline the MAM workflow. The Thermo Exactive Plus instrument was operated in positive ionization mode with the following source conditions: 3.7 kV spray voltage, 250 °C capillary temperature, 250 °C auxiliary gas temperature, 55 S-lens RF level, sheath gas flow rate of 45, and auxiliary gas flow rate of 10. MS data were acquired in full scan mode at 140,000 resolution and with a 3 × 10^6^ AGC target, 200 ms maximum injection time, and 300–2000 *m*/*z* scan range.

MAb2 samples were acquired on two identical LC-MS systems (Thermo Vanquish Horizon UHPLC system coupled to a Thermo Exactive Plus) with the same instrument configuration as described for mAb3. An aliquot of the positive control sample was added for each analytical sequence.

### 4.8. LC-MS MAM Data Analysis

MAb1 data analysis was performed independently in each participating lab. The mAb1 project-specific peptide library was applied to the processed files to match the peptides of interest (unmodified and modified) by their respective RT and *m*/*z* values. A customized validation software plugin was developed in R and implemented in Genedata Expressionist to facilitate the data analysis and review of all library entries of the MAM peptide library simultaneously for all samples within each dataset. Following confirmation that the peak boundaries were correct, the PQA relative abundances were calculated. In case a peak boundary was not optimal for one dataset, a manual peak integration was applied to all samples from the same dataset.

For mAb2, the DSP samples and the three USP sample sets were processed separately for chromatogram smoothing, chemical noise subtraction, RT alignment, peak detection and isotope clustering steps. To apply the same immutable mAb2 MAM peptide library, automated data-driven calibration of library RT values was applied to account for minor chromatographic shifts between the two LC-MS systems. To enable this automated calibration, predefined reference peptides were first annotated with ≤10.0 ppm mass accuracy and a relaxed RT tolerance window of ≤1.0 min. A customized software plugin was developed in R to calculate the differences between the experimental RT values and MAM peptide library RT of these reference peptides. These differences were applied to the library RT entries of dependent peptides. Following the review of peak integration boundaries, and annotation of reference and dependent peptides using the calibrated mAb2 project-specific MAM library with a stringent RT tolerance window ≤ 0.2 min and mass accuracy ≤ 10.0 ppm, the relative abundance of quality attributes was as described above.

MAb3 472 raw files were processed altogether for RT alignment (batch processing). Following a common peak detection and isotopic clustering, the mAb3 MAM peptide library was applied to the 472 processed files together for simultaneous data analysis and data review of peak boundaries as described above, before calculating relative abundances.

An MAM library validation plugin was developed to facilitate data analysis and review by comparing the number of identified entries compared to the MAM peptide library. The validation markers confirm detection (green), absence (red) or ambiguous assignment (yellow). Any misprocessed peak or isotope clusters were edited simultaneously across all samples to ensure consistent relative quantification in the following steps.

For PQA relative quantification, the volumes of each PQA and unmodified peptide were determined as the integral of the intensity values inside the peak boundaries, i.e., intensity values × retention time window × *m*/*z* width, as described before [15,16].

### 4.9. Robustness Assessment

All potential sources of method output variability associated with automated sample preparation for MAM analysis and LC-MS MAM data acquisition were identified using the Ishikawa method for risk assessment. The confirmatory robustness assessments of the MAM method were conducted primarily using a DoE approach, with a fractional factorial design (resolution III) on JMP software v.14 (JMP Statistical Discovery LLC, Cary, NC, USA) to evaluate the main impact of deliberate minor variations in method parameters (factors) on method outputs (responses), while acknowledging that main factor effects may be confounded with two-factor interactions. In cases where DoE was not the most suitable methodology, such as evaluating the incubation temperature or incubation time with the Hamilton liquid handler, a one-factor-at-a-time approach was used. The setpoints for method parameters, as previously described, were applied as the center points in robustness studies. Test ranges for each method parameter were determined with consideration of device systematic errors or instrument qualification limits and were generally larger than these thresholds. The method outputs were selected by evaluating the variability in PQA relative quantification, with automated peak detection using the targeted MAM library being considered. The MAM methods were considered robust if the method output variability fell within accepted limits for quantitative methods by mass spectrometry. In cases where high variability was observed, statistical simulations based on the correlation equation derived from the DoE robustness model were performed to estimate the probability of failure by comparing predicted relative deviation in relation to the predefined ranges of the method parameters.

### 4.10. Experimental Design and DoE Data Analysis

Experimental designs for the two mAbs followed the standard workflow as widely applied in industry, with initially performing a risk assessment to identify the potentially critical process parameters based on existing knowledge. Subsequently, in a study protocol, the appropriate type of study was selected (univariate/multivariate). In case DoEs were used, they were designed and analyzed JMP (versions 16–18). Various type of designs have been applied, depending on prior knowledge on the specific unit operation, parameter interactions and number of parameters. Most commonly, and applicable for the experiments shown, are response surface designs based on the so-called “Custom Designs” in JMP.

### 4.11. Cation-Exchange Chromatography

Samples were diluted in mobile phase A (2.4 mM Tris, 1.4 mM imidazole, 11.6 mM piperazine, pH 5.5) to 3 mg/mL and injected onto a Thermo ProPac WCX-10 (250 × 4 mm, 10 μm). Peaks were separated by applying a linear gradient using mobile phase B (2.4 mM Tris, 11.6 mM piperazine, pH 10.2) at a flow rate of 1 mL/min. UV absorbance was measured at 280 nm.

### 4.12. Hydrophilic Interaction Chromatography

The glycans were enzymatically released from the antibody by peptide N glycosidase F (PNGase F) treatment. The released glycans were derivatized/labeled with the fluorophore 2 aminobenzamide (2-AB) on their reducing termini by reductive amination. After labeling, the excess of free 2-AB reagent was removed by gel filtration. The 2-AB labeled glycans were eluted in aqueous solution. The purified labeled glycans were analyzed by fluorescence detection after separation on an Acquity UPLC BEH Glycan column (1.7 μm, 2.1 × 100 mm, Waters) using ammonium formate at pH 4.4 and acetonitrile.

## Data Availability

The original contributions presented in this study are included in the article. Further inquiries can be directed to the corresponding author.

## Abbreviations

The following abbreviations are used in this manuscript:

ACN	Acetonitrile
ATP	Analytical target profile
CDR	Complementarity determining region
CE-SDS	Capillary electrophoresis sodium dodecyl sulfate
CEX	Cation ion exchange chromatography
CQA	Critical quality attribute
CZE	Capillary zone electrophoresis
CPP	Critical process parameter
DoE	Design of experiments
DSP	Downstream processing
EMA	European Medicines Agency
FDA	U.S. Food and Drug Administration
FLD	Fluorescence detection
GuHCl	Guanidine-HCl
HC	Heavy chain
HILIC	Hydrophilic interaction chromatography
IMHT	Intermediate holding time
IPA	Isopropanol
LC	Light chain
LC-MS	Liquid chromatography–mass spectrometry
mAb	monoclonal antibody
MAM	Multi-attribute method
MS	Mass spectrometry
*m*/*z*	Mass-to-charge ratio
PC	Process characterization
PNGaseF	N-glycosidase F
PTMs	Post-translational modifications
PQA	Product quality attribute
QTPP	Quality target product profile
RP	Reversed-phase chromatography
RSD	Relative standard deviation
RT	Retention time
TFA	Trifluoroacetic acid
USP	Upstream processing
UV	Ultraviolet

## Appendix A

**Table A1:** DoE robustness evaluation of automated sample preparation—variability of chemical modifications. (1) Mean and relative standard deviation (RSD) of 6 center points. (2) Mean and RSD across all the runs in DoE.

Pattern	M253ox [%]	M283ox [%]	M4ox [%]	Deam1 [%]	Deam2 [%]	isoD [%]	Succi1 [%]	Succi2 [%]	Succi3 [%]	Succi4 [%]
Mean_Center **^(^**^1)^	4.0	8.2	1.3	1.5	5.1	17.7	2.8	4.0	36.4	4.0
RSD_Center ^(1)^	2.8	5.1	10.5	5.5	1.8	1.7	2.6	4.5	3.5	1.1
Mean_DoE ^(2)^	4.0	8.2	1.4	1.7	5.2	17.5	2.9	3.8	35.7	3.8
RSD_DoE ^(2)^	3.9	8.5	16.6	58.2	18.3	6.6	32.9	35.0	5.1	25.1

**Table A2:** DoE robustness evaluation of automated sample preparation—ANOVA of chemical modifications. “-”: factor does not significantly impact on the variability of PQAs (*p*-value > 0.01). Pos: significant impact with positive trend (*p*-value < 0.01). Higher factor level leads to increased level of PQAs. Neg: significant impact with negative trend (*p*-value < 0.01). Higher factor level leads to decreased level of PQAs.

Factors	M4ox [%]	Deam1 [%]	Deam2 [%]	Succi1 [%]	Succi2 [%]	Succi4 [%]
Protein conc. [mg/mL]	Neg	-	Pos	-	-	Pos
Denaturation pH	Pos	Pos	Pos	Neg	-	-
DTT conc. [mM]	-	Neg	Neg	Pos	Neg	Neg
Digestion pH	Pos	Pos	Pos	Neg	Pos	Pos

**Table A3:** Estimation of variability of method outputs with significant factors in automated sample preparation. (1) Mean and RSD of the center points. (2) Estimated relative deviation to the Mean_center according to systematic error or specification limit of the significant method parameters.

Pattern	M4ox [%]	Deam1 [%]	Deam2 [%]	Succi 1 [%]	Succi2 [%]	Succi4 [%]
Mean_Center [%] ^(1)^	1.3	1.5	5.1	2.8	4.0	4.0
RSD_Center [%] ^(1)^	10.5	5.5	1.8	2.6	4.5	1.1
Predicted rel. deviation [%] ^(2)^	14.8	50.1	13.1	21.5	11.6	7.7

**Table A4:** DoE robustness evaluation of LC-MS conditions—variability of chemical modifications. (1) Mean and RSD of 12 center points. (2) Mean and RSD across all the runs in DoE.

Pattern	M253ox [%]	M283ox [%]	M4ox [%]	Deam1 [%]	Deam2 [%]	isoD [%]	Succi1 [%]	Succi2 [%]	Succi3 [%]	Succi4 [%]
Mean_Center ^(1)^	4.0	11.9	1.9	1.5	4.7	19.1	3.1	3.7	37.1	4.0
RSD_ Center ^(1)^	1.6	10.0	7.0	4.3	5.5	3.8	10.0	4.0	2.7	6.2
Mean_DoE ^(2)^	4.2	8.7	1.7	1.5	4.9	19.4	3.3	3.7	37.7	4.2
RSD_DoE ^(2)^	6.1	34.8	14.3	6.4	8.0	4.8	9.8	4.7	3.3	8.1

**Table A5:** DoE evaluation of LC-MS conditions—ANOVA of oxidations. Pos: significant impact with positive trend (*p*-value < 0.01). Higher factor level leads to increased level of PQAs.

Factors	M283ox [%]
TFA in mobile phase B [%]	Pos
Column temperature [°C]	Pos
